# Contrasting Inducible Knockdown of the Auxiliary PTEX Component PTEX88 in *P*. *falciparum* and *P*. *berghei* Unmasks a Role in Parasite Virulence

**DOI:** 10.1371/journal.pone.0149296

**Published:** 2016-02-17

**Authors:** Scott A. Chisholm, Emma McHugh, Rachel Lundie, Matthew W. A. Dixon, Sreejoyee Ghosh, Meredith O’Keefe, Leann Tilley, Ming Kalanon, Tania F. de Koning-Ward

**Affiliations:** 1 School of Medicine, Deakin University, Waurn Ponds, Victoria, Australia; 2 Department of Biochemistry and Molecular Biology, Bio21 Institute, Melbourne, Victoria, Australia; 3 The Burnet Institute, Melbourne, Victoria, Australia; Institut national de la santé et de la recherche médicale - Institut Cochin, FRANCE

## Abstract

Pathogenesis of malaria infections is linked to remodeling of erythrocytes, a process dependent on the trafficking of hundreds of parasite-derived proteins into the host erythrocyte. Recent studies have demonstrated that the *Plasmodium* translocon of exported proteins (PTEX) serves as the central gateway for trafficking of these proteins, as inducible knockdown of the core PTEX constituents blocked the trafficking of all classes of cargo into the erythrocyte. However, the role of the auxiliary component PTEX88 in protein export remains less clear. Here we have used inducible knockdown technologies in *P*. *falciparum* and *P*. *berghei* to assess the role of PTEX88 in parasite development and protein export, which reveal that the *in vivo* growth of PTEX88-deficient parasites is hindered. Interestingly, we were unable to link this observation to a general defect in export of a variety of known parasite proteins, suggesting that PTEX88 functions in a different fashion to the core PTEX components. Strikingly, PTEX88-deficient *P*. *berghei* were incapable of causing cerebral malaria despite a robust pro-inflammatory response from the host. These parasites also exhibited a reduced ability to sequester in peripheral tissues and were removed more readily from the circulation by the spleen. In keeping with these findings, PTEX88-deficient *P*. *falciparum*-infected erythrocytes displayed reduced binding to the endothelial cell receptor, CD36. This suggests that PTEX88 likely plays a specific direct or indirect role in mediating parasite sequestration rather than making a universal contribution to the trafficking of all exported proteins.

## Introduction

Malaria remains a significant global health issue, resulting in approximately 584,000 deaths and 200 million clinical cases annually [1]. Clinical symptoms are caused by infection of erythrocytes with parasites of the *Plasmodium* genus, the most virulent of which is *Plasmodium falciparum*. The pathogenicity of *P*. *falciparum* is attributed to its ability to drastically modify the host erythrocyte, both physically and biochemically, primarily by the synthesis and trafficking of hundreds of parasite proteins beyond the parasitophorous vacuole membrane (PVM)(for reviews, see [2,3]). These exported proteins play vital roles in a number of essential parasite processes, including nutrient acquisition and exchange of solutes, and the modification of the erythrocyte to reduce host cell deformability [4–7]. Furthermore, a number of exported proteins facilitate the presentation of adhesins on the outer surface of the host cell [7]. In *P*. *falciparum* these include PfEMP1, which facilitates binding of the infected erythrocyte to the vascular endothelium and thus preventing clearance of parasites through the spleen [8–10] and RIFINs and STEVORs, which bind to uninfected erythrocytes to mediate rosettes [11,12]. The adhesion of infected erythrocytes to blood vessels is also a major contributor to the pathogenesis and severity of malaria infections [11,13].

Most of the 450 proteins exported by *P*. *falciparum* into the host erythrocyte contain a conserved ‘PEXEL’ or ‘host targeting’ (HT) motif [14,15]. However, some proteins lacking a PEXEL motif can also be exported and as these so called PEXEL-negative exported proteins (PNEPs) do not share any primary sequence information, it is difficult to predict how many PNEPs are exported by *P*. *falciparum* [16]. Protein export into the host erythrocyte also occurs in other *Plasmodium* species and while the total number of PEXEL proteins is smaller relative to that found in *P*. *falciparum*, other *Plasmodium* species possess a large number of PNEPs [16–18]. Irrespective of the difference in size of the exportomes of the different *Plasmodium* spp, the conservation of the PEXEL motif, the presence of PNEPs, as well as the observation that a number of exported proteins reside in membranous structures in the host cell cytoplasm in both human and rodent malaria species [19–23] suggest that the mechanisms of protein export is also conserved across the genus.

Residing at the PVM in *P*. *falciparum* is a macromolecular complex termed the *Plasmodium* Translocon of EXported proteins (PTEX)[24]. PTEX was originally predicted to facilitate trafficking of PEXEL-containing proteins across the PVM, but more recent studies have validated that PTEX is responsible for the trafficking of PEXEL proteins as well as PNEPs into the host cell, with cargo including soluble and transmembrane domain-containing proteins [25,26]. PTEX comprises of five known components, and the genes encoding these components are conserved amongst, but unique to members of the *Plasmodium* genus. More recently, the five PTEX components have also been shown to form a complex at the PVM of the rodent malaria species, *P*. *berghei* [27].

The translocon’s putative energy source is ATP, which drives the PTEX component HSP101, a AAA+ ATPase. Conditional knockdown of HSP101 function results in a striking block in protein export and parasite lethality, consistent with this alleged role for HSP101 [25,26]. Another component of PTEX is a known PVM resident protein, EXP2 [28,29] and structural modeling of this protein suggests it forms a pore through the PVM, thereby allowing the passage of exported cargo [24,30]. Recently, EXP2 was shown to be able to complement the function of GRA17, a dense granule protein in the related Apicomplexan parasite and which is thought to form large non-selective pores [31]. The inability to knockout *exp2* in the blood-stages and the reduced patency of FLP-*FRT*-conditional *exp2* mutants in mice is in keeping with its predicted pivotal role in PTEX [27,32]. PTEX also comprises thioredoxin 2 (TRX2); whilst not essential to *P*. *berghei* survival in mice [27,33], TRX2 may play a role in PTEX function by aiding in the unfolding of exported cargo so that they are competent for export or TRX2 may help to regulate the formation of disulphide bonds in the PTEX complex via redox activity. The other two PTEX components, PTEX150 and PTEX88 have no conserved motifs or show any homology to other known proteins to predict likely function. However, inducible knockdown of PTEX150 leads to loss in protein export and parasite lethality [25], which would explain why attempts to genetically delete *ptex150* have been unsuccessful [24,27,33]. Since PTEX150 has also been shown to bind tightly to HSP101 and EXP2 [30], it has been proposed that PTEX150 has a structural role in PTEX function. [27]. In contrast, *ptex88* could be successfully deleted in *P*. *berghei* [33]. The *ptex88* knockout parasites exhibited reduced sequestration and virulence and the infected mice displayed splenomegaly. These phenotypes could not be linked to alterations in protein export, which led the authors to cast doubt on the role of PTEX88 in the protein translocation process [34]. However, PTEX88 localises to the PVM and immunoprecipitation experiments have confirmed that PTEX88 interacts with all other known components of the translocon, indicating that PTEX88 is indeed a genuine component of the translocon [24,27].

Since *P*. *berghei* and *P*. *falciparum* exhibit little overlap in their exportomes, we sought to determine, therefore, whether knocking down the expression of PTEX88 in *P*. *falciparum* could reveal a role for this component in protein export. In parallel, we also conditionally depleted PTEX88 in *P*. *berghei* to rule out the possibility that loss of the *ptex88* gene leads to compensatory export mechanisms given that studies in the related Apicomplexan parasite, *Toxoplasma gondii*, have revealed they can exhibit phenotypic plasticity to preserve function [35]. We show that while loss of PTEX88 in *P*. *falciparum* does not alter the maintenance of normal parasite growth *in vitro*, it reduces the cytoadhesive capacity of infected erythrocytes. Our findings also corroborate the observations by Matz et al [34], demonstrating that depletion of PTEX88 in *P*. *berghei* impairs parasite growth *in vivo* and alters the ability of the parasite to sequester in peripheral tissues. We confirm that PTEX88 does not appear to be required in *P*. *berghei* for export of a variety of known exported proteins and we further demonstrate that this is also the case in *P*. *falciparum*. We show that mice infected with parasites depleted of PTEX88 do not succumb to cerebral malaria but reveal that this phenotype could not be linked to an alteration in the pro-inflammatory immune response from the host and instead appears to result from reduced cytoadhesion of infected erythrocytes. These observations suggest that PTEX88 does not function in the same manner as the core PTEX components in the process of protein export and is more likely aiding in particular parasite processes such as sequestration.

## Materials and Methods

### Ethics approval

All experiments involving the use of animals were performed in strict accordance with the recommendations of the Australian Government and the National Health and Medical Research Council Australian code of practice for the care and use of animals for scientific purposes. The protocols were approved by the Deakin University Animal Welfare Committee (approval number G37/2013 and G16/2014).

### Plasmid constructs

To create the targeting construct for knocking down the expression of the endogenous *P*. *falciparum ptex88* gene, the glucosamine-inducible *glmS* ribozyme sequence and the *P*. *berghei dhfr-ts* 3' UTR were excised from pTEX150-HA-glmS [25] with *Pst*I and *Sac*I and cloned into the corresponding restriction sites of pPfTEX88-HA/Str [27]. To create the plasmid used to engineer the PbPTEX88 inducible knockdown (iKD) line, 1.2 Kb of sequence immediately upstream of the PTEX88 start codon was PCR amplified from *P*. *berghei* ANKA gDNA using the primers O453F and O454R (see S1 Table for oligonucleotide sequences). The resulting product was ligated into *Nhe*I and *Bss*HII sites of pPRF-TRAD4-Tet07-HAPRF-hDHFR [36] that had been modified to include a *Bss*HII restriction enzyme site between the profilin 5' UTR and TRAD4 sequence. Following successful ligation, the first 1.2 Kb of the PbPTEX88 coding sequence was amplified by PCR using the primers O451F and O452R and cloned into the intermediate vector using the *Pst*I and *Nhe*I restriction to create the final targeting vector, termed pTRAD4-iPTEX88. Before transfection into *P*. *berghei* ANKA parasites, pTRAD4-iPTEX88 was linearized with *Nhe*I to drive integration into the *ptex88* locus.

### Parasites and transfection

Blood-stage *P*. *falciparum* 3D7 was cultured continuously [37] and transfected as previously described [38]. Transgenic parasites were selected with 2.5 nM WR99210 (Jacobus). For the generation of *P*. *berghei* transgenic parasites, the reference clone15cy1 from the *P*. *berghei* ANKA strain was used. Transfection of parasites and selection of the transgenic parasites was performed as previously described [39]. Briefly, Nycodenz-purified *P*. *berghei* schizonts were prepared for transfection and DNA constructs were introduced using the Nucleofector^®^ electroporation device (AMAXA). The resulting DNA mixture was injected intravenously into 6- to 8-week-old BALB/c mice and drug selection of genetically transformed parasites commenced at day one post transfection by administration of pyrimethamine (0.07 mg/mL) in the drinking water of mice.

### Nucleic acid analysis

The genotypes of the PfPTEX88-HA, PTEX88-HA-glmS and PbPTEX88 iKD transgenic lines were all confirmed by diagnostic PCRs. Integration of plasmids into the endogenous *pfptex88* locus was confirmed using oligonucleotide primer sets O295F/O145R and O295F/O276. Integration of the targeting construct into the endogenous *pbptex88* locus was confirmed using oligonucleotide primer sets O453F/O162R and O213F/SH4R.

To detect transcripts in *P*. *berghei* ANKA parasites by qRT-PCR, RNA was extracted from blood stage parasites, using TRIsure^™^ reagent (Bioline). cDNA was then made using the iScript™ reverse transcription supermix (Biorad) according to the manufacturer’s instructions. cDNA (or gDNA as a control) was used in PCR reactions using oligonucleotides to *ptex88* (O587F/O588R) or *gapdh* (O567F/O568R). The expression levels of *ptex88* was normalised against the *gapdh* house-keeping gene, with gene expression values calculated based on the 2^ΔΔCt^ method.

### Parasite growth analysis

For *P*. *falciparum* assays, parasites were sorbitol synchronized and allowed to reinvade new erythrocytes. In the subsequent cycle when parasites were at trophozoite stage, glucosamine (GlcN) was added to a final concentration of 0.1 mM, 0.3 mM, 1.0 mM or 2.5 mM, with 0 mM GlcN serving as the negative control. Parasite growth 48 hrs after the addition of GlcN was assessed using the pLDH assay method to measure levels of the parasite enzyme lactate dehydrogenase [40].

To assess the effect of the PTEX88 knockdown on *P*. *berghei* parasite growth, female Balb/c mice between 5–6 weeks of age were randomized into groups of five mice per experiment and then given drinking water containing either 0.2mg/ml ATc (Sigma) made in 5% (w/v) sucrose or 5% sucrose only as a vehicle control. After 48 hours pre-treatment, mice were infected intraperitoneally (i.p) with 1x10^6^ parasitized PbPTEX88 iKD erythrocytes. From 3 days post infection, parasitemias were monitored daily by Giemsa-stained tail blood smears. Once the parasitemias reached >20%, mice were humanely culled. Parasitemias were determined by counting a minimum of 1000 erythrocytes. Statistical analysis was performed using a students *t*-test. To establish synchronous *P*. *berghei* infections for more precise comparisons of parasite growth, infected blood harvested from donor mice was cultured overnight in RPMI/20% FCS. Schizonts were purified on Nycodenz (Axis-Shield) and then intravenously injected into two mice per group (n = 2 experiments) that had either been pre-treated with ATc or vehicle control. Tail blood smears were made every three hours and stained with Giemsa. At 12 hrs post infection (hpi), infected blood was harvested by cardiac bleed and cultured *in vitro* in the presence or absence of ATc, with smears again taken every three hours to monitor parasite growth, schizont development and merozoite formation. At each time point, the total parasitemia was determined as was the stage of parasite growth.

### Analysis of parasite virulence and parasite burden

Female C57/Bl6 mice at 6 weeks of age were either pretreated with ATc (0.2mg/mL) or vehicle control prior to i.p infection with 1x10^6^ PbPTEX88 iKD parasitized erythrocytes. To assess parasite virulence, mice were monitored for cerebral malaria symptoms including ataxia and inability to self-right from day 4 post-infection. Mice were humanely culled when displaying cerebral malaria symptoms or when the parasitemia exceeded 20%. Statistical analysis of parasite survival was performed using a Wilcoxon log-rank test (n = 6 mice per group). To determine the parasite burden by qRT-PCR, mice (n = 5) were sacrificed at day 5 post-infection and perfused intracardially with isotonic NaCl solution. Adipose tissue, lung and spleen were homogenized in TRIsure^™^ reagent (Bioline) for RNA extraction.

### Immune analysis

Female C57/Bl6 mice were pretreated with ATc (0.2mg/mL) or vehicle control (n = 5 per group) and infected i.p the subsequent day with 1x10^6^ PbPTEX88 iKD parasitized erythrocytes. At day 5 post-infection the spleens and serum were collected. To analyse the production of cytokines and chemokines in the spleen, lysates were prepared as previously described [41]. Briefly, a small portion of each spleen was harvested into ice-cold RPMI containing cOmplete™ protease inhibitor cocktail (Roche). Single cell suspensions were obtained following passage through 70μm cell strainers, and cells were lysed using 0.5% v/v Triton X-100 for 15 mins on ice. After centrifugation at 14000 x *g* for 5 mins, supernantants were immediately snap-frozen and stored at -80°C. Spleen lysates and serum samples were then analysed using the ProcartaPlex Mouse Cytokine and Chemokine Panel 1 kit (26 plex, eBioscience) according to manufacturer’s instructions. For isolation and flow cytometric analysis of immune cell populations in the spleen, a portion of each spleen was harvested into ice-cold RPMI containing 2% FCS, mechanically chopped, digested with collagenase and DNase and treated with EDTA as previously described [42]. Half of the single cell suspension was enriched for dendritic cells (DCs) using a 1.077g/cm^3^ density centrifugation (Nycodenz) and stained with fluorochrome-conjugated monoclonal antibodies (mAbs) against CD11c, MHC class II, CD8, CD172a, CD45R, CD40 and CD86. Splenic DCs were gated as NK1.1^-^CD49b^-^CD3^-^CD19^-^Ly6G^-^CD11c^+^MHC class II^+^ cells, and then further subdivided into CD8^+^ or CD8^-^ (CD172a^+^) conventional DC subsets, and CD45R^+^ plasmacytoid DCs. The remaining single cell suspension was treated with erythrocyte lysis buffer and stained with combinations of fluorochrome-conjugated mAbs against CD3, CD4 or CD8 (T cells), CD45R and CD19 (B cells), NK1.1 and CD49b (NK cells), CD11c and MHC class II (DCs), or CD11b, CD64, Ly6C and Ly6G (macrophages, monocytes and neutrophils). Flow cytometry was performed on a FACS Canto II (BD Biosciences), excluding doublets and propidium iodide (PI)-positive dead cells, and data were analysed using FlowJo software (v9.4.10; Tree Star Inc., USA).

### Indirect IFA

Thin smears of infected erythrocytes were fixed with ice cold 90% acetone/10% methanol for 1 minute. Cells were then blocked in 1% (w/v) BSA/PBS for 1 hour. All antibody incubations were performed in 0.5% (w/v) BSA/PBS. Primary antibodies for *P*. *falciparum* were used at the following concentrations: rabbit anti-EXP2 1:500, mouse anti-EXP2 1:500, mouse anti-RESA 1:1000, mouse anti-SBP1 1:1000, rabbit anti-KAHRP 1:1000, rabbit anti-PfEMP1 acidic terminal segment (ATS) 1:750, rat anti-RIF50 1:200 [43] and rabbit anti-STEVOR (PFL2610w) 1:500. For *P*. *berghei*, primary antibodies against PbANKA_122900 and PbANKA_114540 were used at 1:500. After one hour incubation in primary antibody, cells were washed three times in PBS and then incubated with the appropriate AlexaFluor 488/568-conjugated secondary antibody (1:2000) for 1 hour. Cells were washed three times in PBS, and mounted in Vectashield mounting medium containing the nuclear stain 4',6-diamidino-2-phenylindole (DAPI) (VectorLabs). Images were taken on an Olympus IX71 microscope and images were processed using ImageJ v1.46r.

Image scoring was performed as described previously [25]. Briefly, for all images, the cell area of whole infected erythrocyte was selected and the mean fluorescence intensity was obtained by means of the ‘Measure’ function. To score the degree of RESA, KAHRP and STEVOR export, the circumference of the infected erythrocyte was first traced around, followed by the parasite (denoted by EXP2 and DAPI staining) to exclude it from subsequent export analysis. The mean fluorescence intensity of RESA was then quantified as above. The labeling of SBP1 and RIFINs in the infected erythrocyte cytosol were similarly traced and the punctate Maurer’s clefts were counted, using the ‘FindMaxima’ function set to a noise tolerance of 200 and ‘Point Selection’ output type. All measurements were graphed using GraphPad Prism.

### Measurement of global protein export

For analysis of *P*. *berghei* antigens on the infected erythrocte surface using fluorescence activated cell sorting (FACS), blood collected from the tail vein of *P*. *berghei* infected mice was washed briefly in RPMI and then blocked for 1 h in 1% casein in RPMI. Erythrocytes were then incubated for 1 h with serum harvested from either *P*. *berghei* semi-immune or non-immune (pre-bleed) mice [25], which was diluted 1:20 in blocking solution. After three washes with block solution, cells were incubated for 1 h with goat anti-mouse IgG AlexaFlour 647 (1:2000; Invitrogen), washed a further three times and then incubated for 5 min in SYBR-Green (Invitrogen) diluted 1:2000 in blocking solution. A further three washing steps were performed, after which the cell preparation was analysed with a FACS Canto II machine (BD Biosciences).

### CD36 binding assay

Recombinant CD36 (125 μg ml^-1^ in PBS) was loaded into culture chambers (iBIDI μ-Slide I) and incubated overnight at 4°C, as previously described [44]. PTEX88-glmS parasites were knocked down by the addition of 2.5 mM GlcN. Untreated and GlcN-treated parasites were synchronised to a 4-hour window. At 26–30 h post-invasion, parasites (1% hematocrit, 3% parasitemia) were resuspended in bicarbonate-free RPMI-HEPES and flowed through the chambers at 0.1 Pa using a Harvard Elite 11 Syringe Pump. Assays were performed at 37°C and were visualised on a DeltaVision DV Elite Restorative Widefield Deconvolution Imaging System (Applied Precision) using a 60X objective. Parasites were flowed through the chamber for 5 min then washed for 10 min in bicarbonate-free RPMI-HEPES. The number of bound cells was then counted for the same 10 randomly chosen fields each experiment.

### Trypsin cleavage of surface proteins

Mature trophozoite stage parasites were enriched from culture by Percoll purification. Purified parasites (~10^6^ iRBCs) were treated with PBS alone or with TPCK-treated trypsin in PBS (Sigma, 1mg/ml), for one hour at 37°C. Following incubation the samples were incubated with soybean trypsin inhibitor (5 mg/ml) for 20min at room temperature to inactivate trypsin. Samples were lysed with 1% Triton X-100 on ice for 20 min and centrifuged at 16 000 *g* for 5 min. The pellet fraction was solubilized for 20 min at room temperature in 2% SDS. Parasite extracts were separated on 3–8% Tris-acetate gels (Invitrogen), transferred onto nitro- cellulose membranes and probed with monoclonal anti- PfEMP1 acidic terminal segment (ATS) (1:100) primary and anti-mouse-HRP (Promega) secondary antibodies.

## Results

### Generating PTEX88-glmS parasites

**Fig 1:**
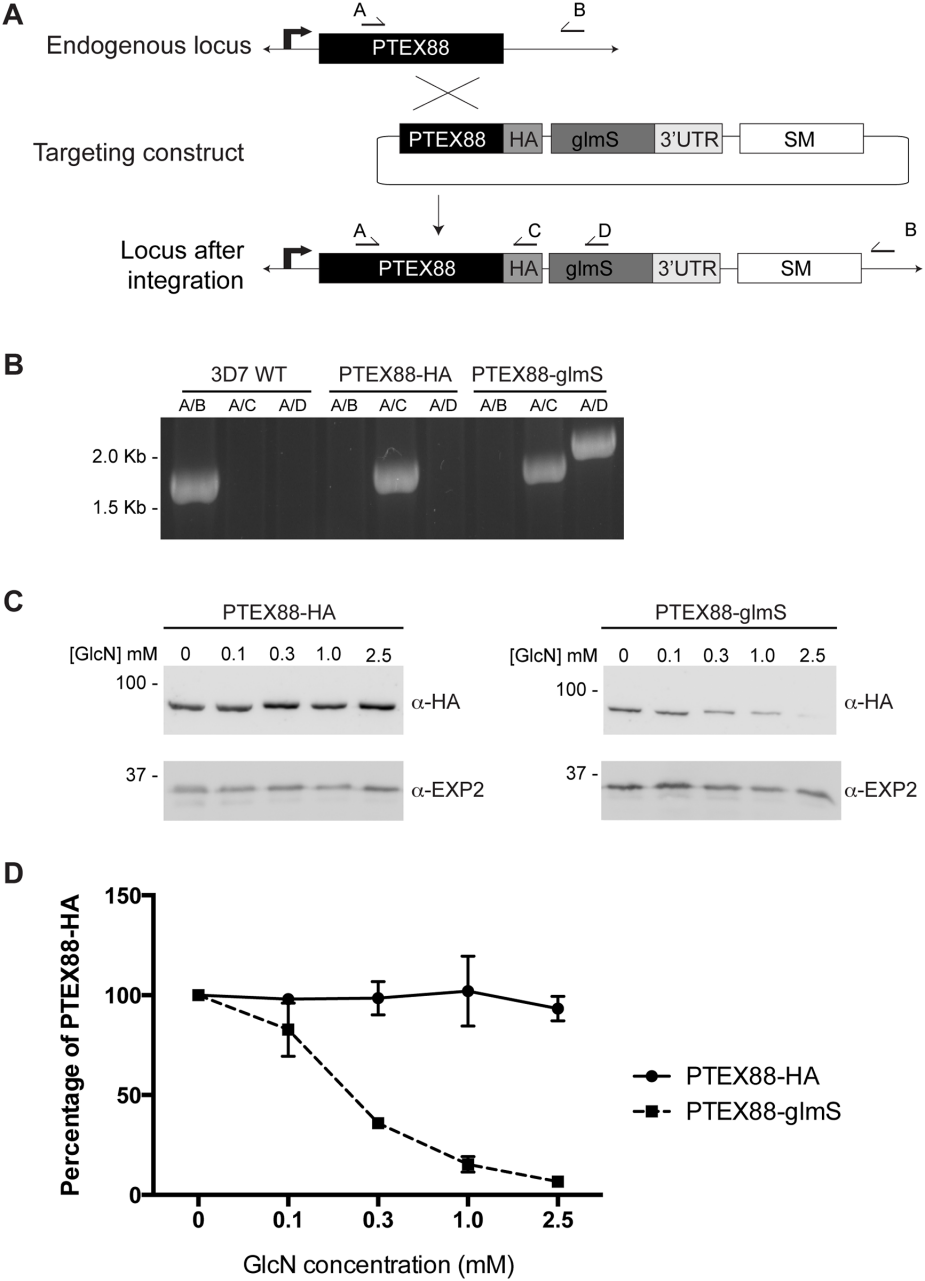
Generation of a PTEX88 knockdown line in *Plasmodium falciparum*. **A**: Schematic of targeting construct designed to integrate into the endogenous *ptex88* locus by single crossover recombination. Arrows indicate binding sites for diagnostic PCR primers. SM, selectable marker; HA, haemaglutinin epitope tag; glmS, glmS ribozyme. **B**: Diagnostic PCR on wildtype (WT) and transgenic genomic DNA using the indicated primer combinations to test for integration of the targeting construct. Absence of a product using primer combination A/B in PTEX88-HA and PTEX88-glmS gDNA indicates these parasite lines are clonal. **C**: Representative Western blot showing levels of tagged PTEX88 in PTEX88-HA control parasites and PTEX88-glmS parasites after incubation with the indicated concentration of glucosamine. EXP2 was used as a loading control. GlcN, glucosamine. **D**: Densitometry performed on Western blots to quantify the protein levels of tagged PTEX88 in the control PTEX88-HA line and the PTEX88-glmS line. The protein level of PTEX88 does not decrease in the control PTEX88-HA line but decreases in the PTEX88-glmS with increasing GlcN concentrations by up to 90% when compared to the sample without GlcN (n = 4).

To assess the role of PTEX88 in parasite growth and protein export in *P*. *falciparum*, an approach utilising the riboswitch system to conditionally regulate the expression of *P*. *falciparum* PTEX88 was undertaken. In this case, single-crossover recombination of the construct pPTEX88-glmS into the *ptex88* locus readily yielded a transgenic parasite line subsequently termed PTEX88-glmS, in which the gene encoding PTEX88 had been modified to incorporate a triple hemagglutinin epitope tag at its C-terminal end and the glucosamine inducible *glmS* ribozyme within its 3' untranslated region. (Fig 1A). Diagnostic PCR confirmed the targeting construct had integrated into the *ptex88* locus as expected and that the PTEX88-glmS parasite population was clonal (Fig 1B).

The ability of PTEX88 to be regulated in the PTEX88-glmS line was then tested by culturing the parasites in increasing concentrations of glucosamine for 48 h. As a control, PTEX88 expression was also analysed in PTEX88-HA, a parasite line lacking the *glmS* ribozyme [27] and thus should not be affected by the addition of glucosamine. Protein material extracted from treated parasites was analysed by Western blot using anti-HA antibodies to detect PTEX88 expression, with EXP2 serving as a loading control (Fig 1C). Densitometry analysis of the resultant blots showed a dose-dependent knockdown of PTEX88-glmS, with 90% knockdown of PTEX88-glmS expression in parasites treated with 2.5 mM glucosamine. In contrast, expression of PfPTEX88-HA remained unaffected by the presence of glucosamine, even at the higher concentration of 2.5 mM (Fig 1D).

### PTEX88 knockdown in *P*. *falciparum* has no observable effect on protein export

**Fig 2:**
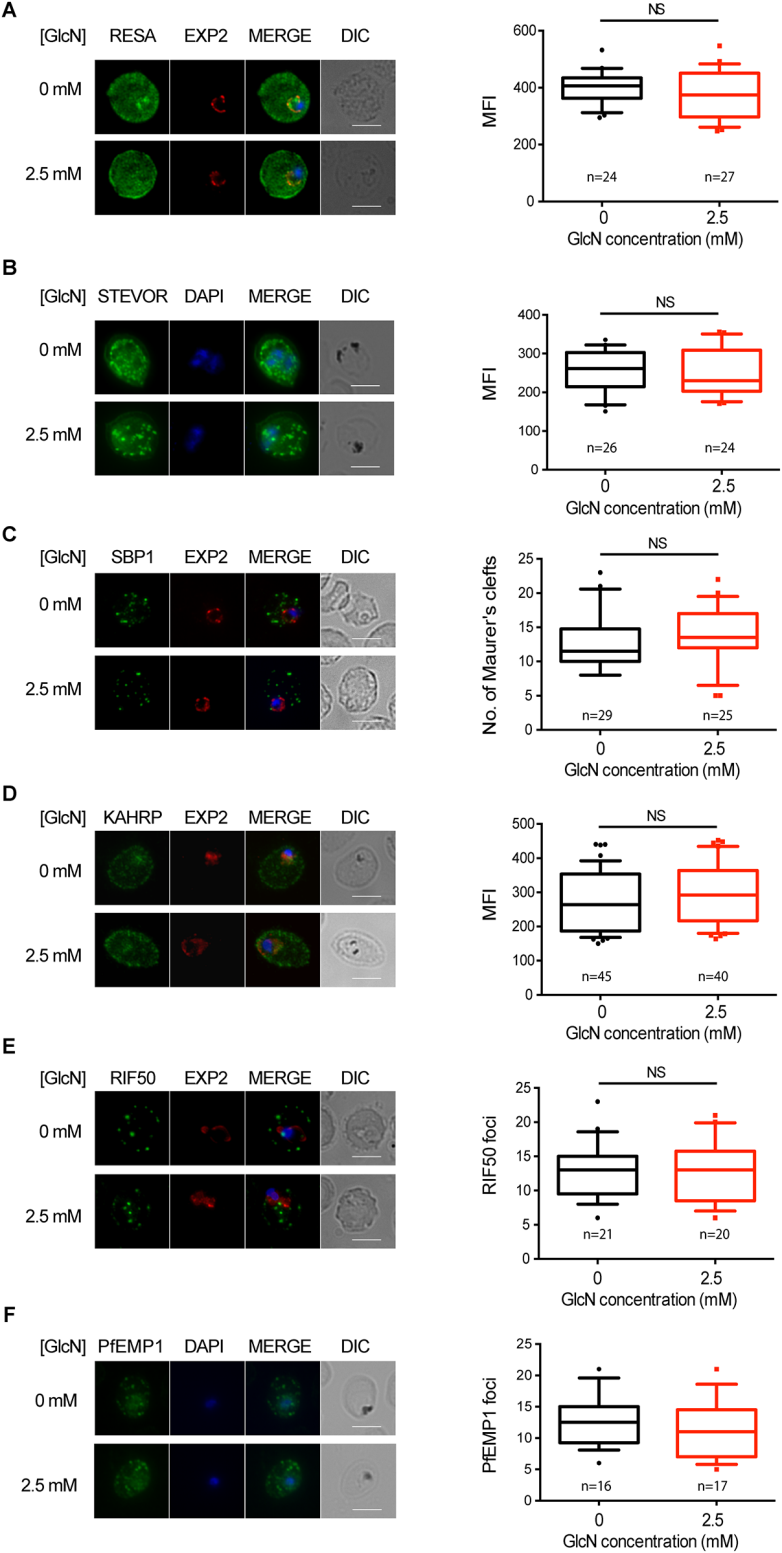
Effect of PTEX88 knockdown on *P*. *falciparum* protein export. Immunofluorescence assays showing that exported proteins **A**: RESA, **B**: STEVOR, **C**: SBP1, **D**: KAHRP, **E**: RIF50, **F**: PfEMP1 are still exported after knockdown of PTEX88. Mean fluorescence intensity was calculated for RESA, STEVOR and KAHRP, whilst the number of Maurer’s clefts was calculated for SBP1. Boxes and whiskers delineate 25–75^th^ and 10–90 percentiles, respectively.

Previously it had been shown that knockdown of the essential PTEX components PTEX150 and HSP101 resulted in a failure to export a range of different protein cargo [25,26]. As PTEX88 has also been confirmed to be a *bona-fide* constituent of PTEX [27], the effect of PTEX88 knockdown on protein export was assessed on fixed parasites labeled with antibodies against various exported proteins. This included RESA (an early expressed PEXEL protein), STEVOR (a PEXEL protein containing transmembrane domains), skeleton binding protein 1 (SBP1; a PNEP), KAHRP (a soluble PEXEL protein), RIF50 (a PEXEL protein containing transmembrane domains) and PfEMP1 (a PNEP containing a transmembrane domain that is exported to the erythrocyte surface (Fig 2A–2F), with samples harvested at time points post-infection when these proteins are maximally exported [25]. Immunofluorescence analysis (IFA) revealed that export of all of these proteins was not obviously affected when expression of PTEX88 was knocked down by the addition of 2.5 mM glucosamine (Fig 2A–2F, left panels) and this was confirmed by quantification using previously described methods [25](Fig 2A–2F, right panels).

### Knockdown of PTEX88 in *P*. *falciparum* has no effect on parasite growth

**Fig 3:**
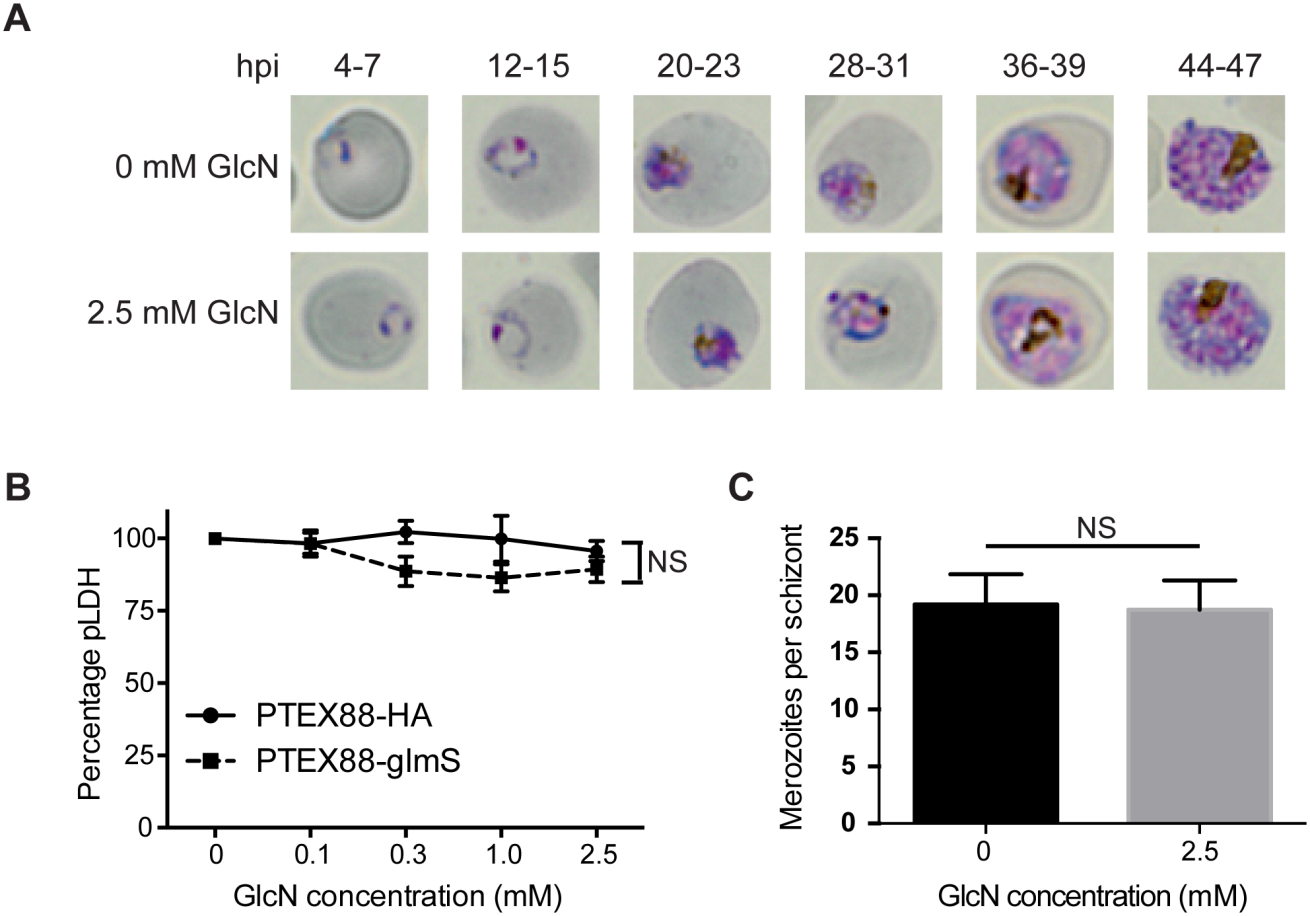
Knockdown of PTEX88 does not affect the growth of *P*. *falciparum*. **A**: Giemsa stained blood smears of *P*. falciparum-infected erythrocytes shows no effect on parasite growth in the PTEX88-glmS line when treated with 2.5mM glucosamine relative to the untreated parasites. **B**: Lactate dehydrogenase (LDH) activity assay of infected erythrocytes assayed approximately 24 hours after parasite invasion indicate that growth of control parasites and PTEX88-glmS parasites is unaffected upon glucosamine treatment. (n = 4). **C**: Reduction of PTEX88 protein levels with 2.5 mM glucosamine does not affect the merozoite formation with a schizont (n = 15 per group).

The exported proteins that were analysed above are proteins that have been linked to parasite virulence and not likely to contribute to parasite survival *in vitro*. However, conditional regulation of *P*. *falciparum* PTEX150 and HSP101 leads to severe defects in parasite growth demonstrating that at least some exported proteins contribute to parasite survival. This is in keeping with the findings of a large scale gene knockout approach in *P*. *falciparum*, which revealed ~25% of exported proteins could not be targeted for disruption and are, therefore, likely to be essential [45]. Thus to explore the effect of PTEX88 knockdown on the growth of parasites, PTEX88-glmS was synchronised to within a 3 h window after treatment with either vehicle control or 2.5 mM glucosamine. Giemsa-stained blood smears were then made at 8 h intervals to observe parasite morphology across two cell cycles. There was no observable difference in morphology of PTEX88-glmS grown in either the presence or absence of glucosamine across the entire blood-stage life cycle (Fig 3A), suggesting that even a 90% reduction in PTEX88 expression does not have an adverse affect on parasite growth. To corroborate this growth result, parasite lactate dehydrogenase (pLDH) assays were performed on glucosamine-treated parasites to assess growth quantitatively. However, the pLDH levels of PTEX88-glmS were not significantly reduced when compared to PfPTEX88-HA grown in glucosamine or PTEX88-glmS grown in the absence of glucosamine (Fig 3B). There was also no significant difference in the number of merozoites generated at the completion of the asexual lifecycle (Fig 3C). Although a conditional PfPTEX88-HA mutant cannot be considered a null mutant, these results suggest that PTEX88 is not crucial for maintaining normal parasite growth under routine culturing conditions of *P*. *falciparum in vitro*.

### Generation of an ATc regulatable PTEX88 inducible knockdown in *P*. *berghei*

**Fig 4:**
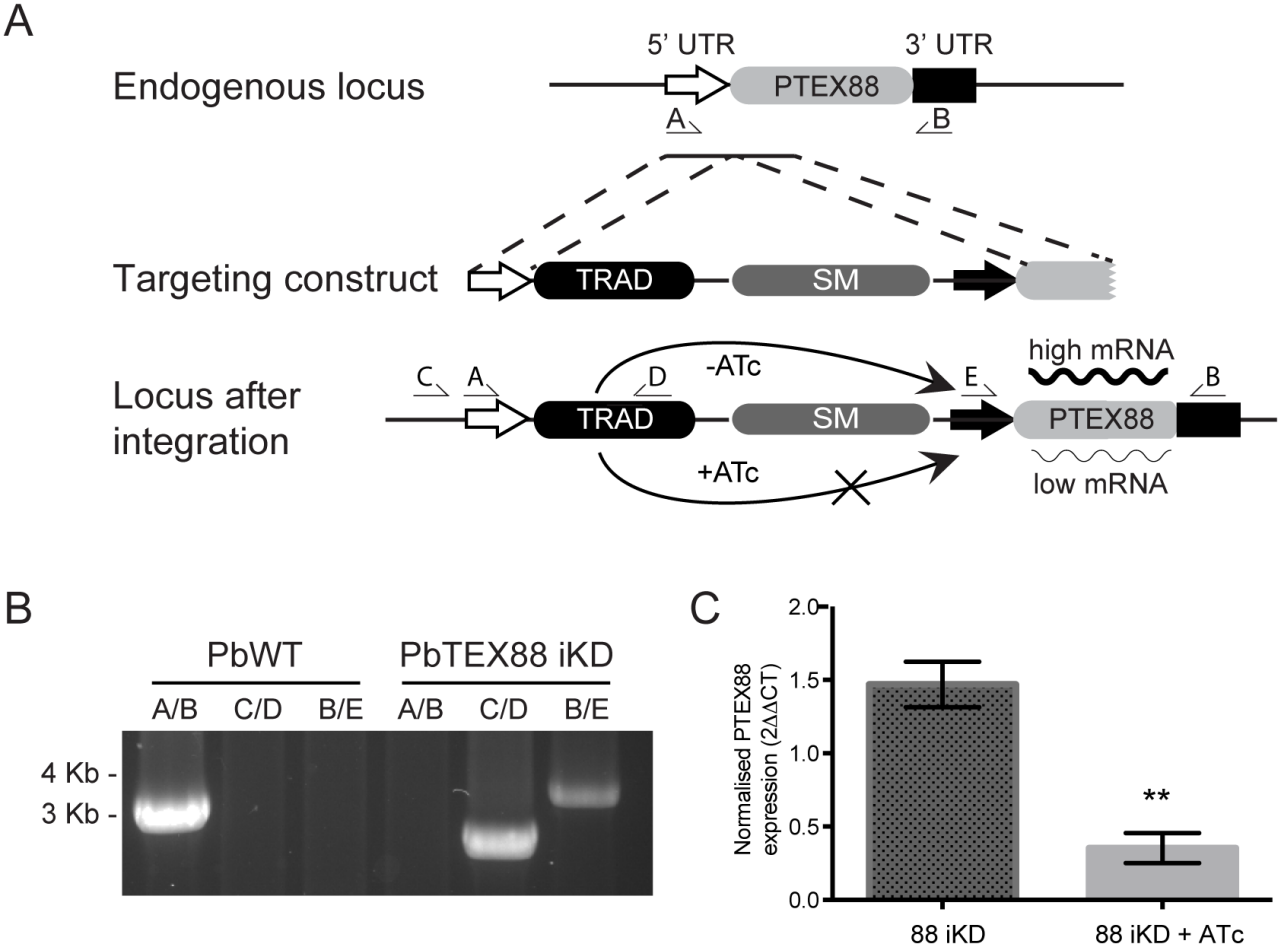
Generation of an inducible PTEX88 knockdown line in *P*. *berghei*. **A**: Schematic representation of the targeting construct used to generate the PbPTEX88 iKD line. Successful integration results in the full *ptex88* gene being under control of a minimal promoter (solid black arrow) and the TRAD transactivator under the *ptex88* 5' UTR. Arrows indicate binding sites for diagnostic PCR primers. **B**: Diagnostic PCR on parasite genomic DNA using indicated primer combinations demonstrate correct integration of the targeting construct. Absence of a product using primer combination A/B in PbPTEX88 iKD gDNA indicates this line is clonal. **C**: Quantitative RT-PCR on parasite material isolated from PbPTEX88 iKD parasites shows a significant knockdown of *ptex88* transcript in parasites exposed to ATc.

Since proteins exported by *Plasmodium* have diverse functions, some of which may not play any significant role to parasite survival when malaria parasites are grown in an *in vitro* context, we next analysed the consequences of knocking down expression of PTEX88 in *P*. *berghei*. In this case, placement of *ptex88* under the control of an anhydrotetracycline (ATc) controlled transactivator would enable the acute regulation of PTEX88 expression through the administration of ATc to the drinking water of mice (Fig 4A). This contrasts to a PTEX88 gene knockout [34] as parasites do not have the capacity to adapt to the absence of the gene within one to two parasite cycles. Verification that this transgenic parasite line, termed PbPTEX88 iKD, had been correctly engineered was obtained by diagnostic PCR, which also confirmed that the parasite population was clonal (Fig 4B). Quantitative RT-PCR (qRT-PCR) was used to quantify the reduction of *ptex88* transcript after ATc treatment since repeated attempts to generate antibodies to PTEX88 failed. The qRT-PCR revealed that *ptex88* expression could be successfully regulated by ATc, with an average of 4-fold reduction in *ptex88* transcript observed (Fig 4C). In contrast, transcript levels of *gapdh* remained unaffected by ATc treatment.

### Protein export in *P*. *berghei* is largely unaffected by knockdown of PTEX88

**Fig 5:**
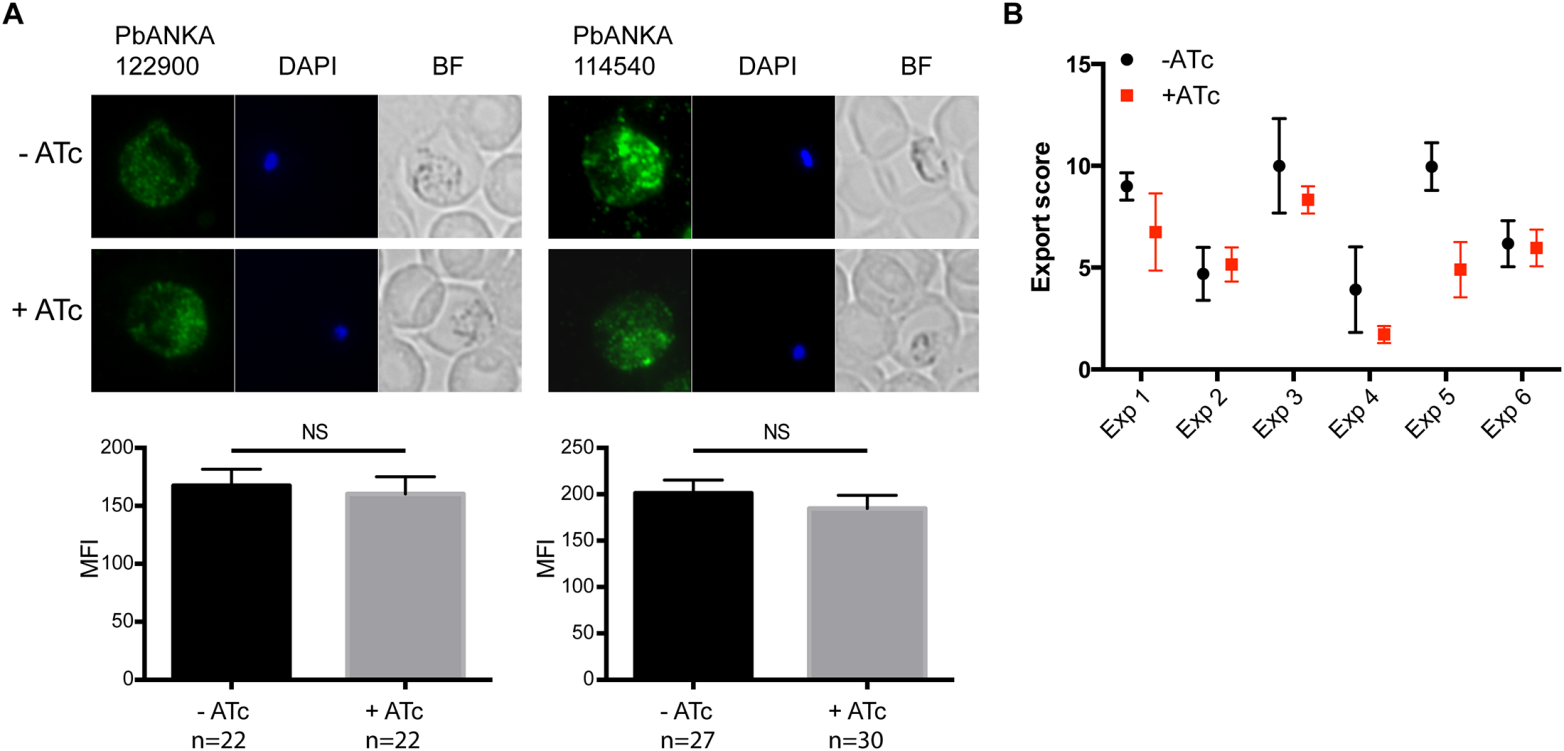
Effect of PTEX88 knockdown on protein export in *P*. *berghei*. **A**: Immunofluorescence assays showing the *P*. *berghei* proteins PBANKA_114540 and PBANKA_122900 are still exported after conditional depletion of PTEX88 in PbPTEX88iKD parasites using ATc. Export was measured by calculating MFI, shown as the mean + SEM. **B**: Surface labelling of parasite antigens on PbPTEX88 iKD parasites harvested between 4 or 5 days post infection compared with infected erythrocytes not exposed to ATc as measured by FACS (n = 6). A mixed ANOVA was used to test for statistical significance.

Having demonstrated that the expression of *P*. *berghei* PTEX88 could be controlled by the addition of ATc, we next examined whether conditionally regulating PTEX88 expression affected the acute ability of parasites to export their proteins. Parasites were fixed and labeled with antisera against *P*. *berghei* proteins PbANKA_122900 and PbANKA_114540, export of which was previously shown to be affected in *P*. *berghei* parasites conditionally depleted of HSP101 [25]. However, quantification of IFA images revealed there was no significant decrease in the export of either of these proteins when PTEX88 expression was reduced (Fig 5A). We also used a FACS-based method [25] to quantitate parasite-encoded proteins exposed on the surface of infected erythrocytes harvested from mice treated with ATc or vehicle control (3 mice per group). Although we observed that parasites treated with ATc displayed less surface antigens than control parasites in some experiments, there was no significant difference overall in protein export between treatment groups across six independent experiments using a mixed ANOVA test (p = 0.06),(Fig 5B).

### Knockdown of PTEX88 increases parasite clearance *in vivo* and influences virulence and binding to CD36

**Fig 6:**
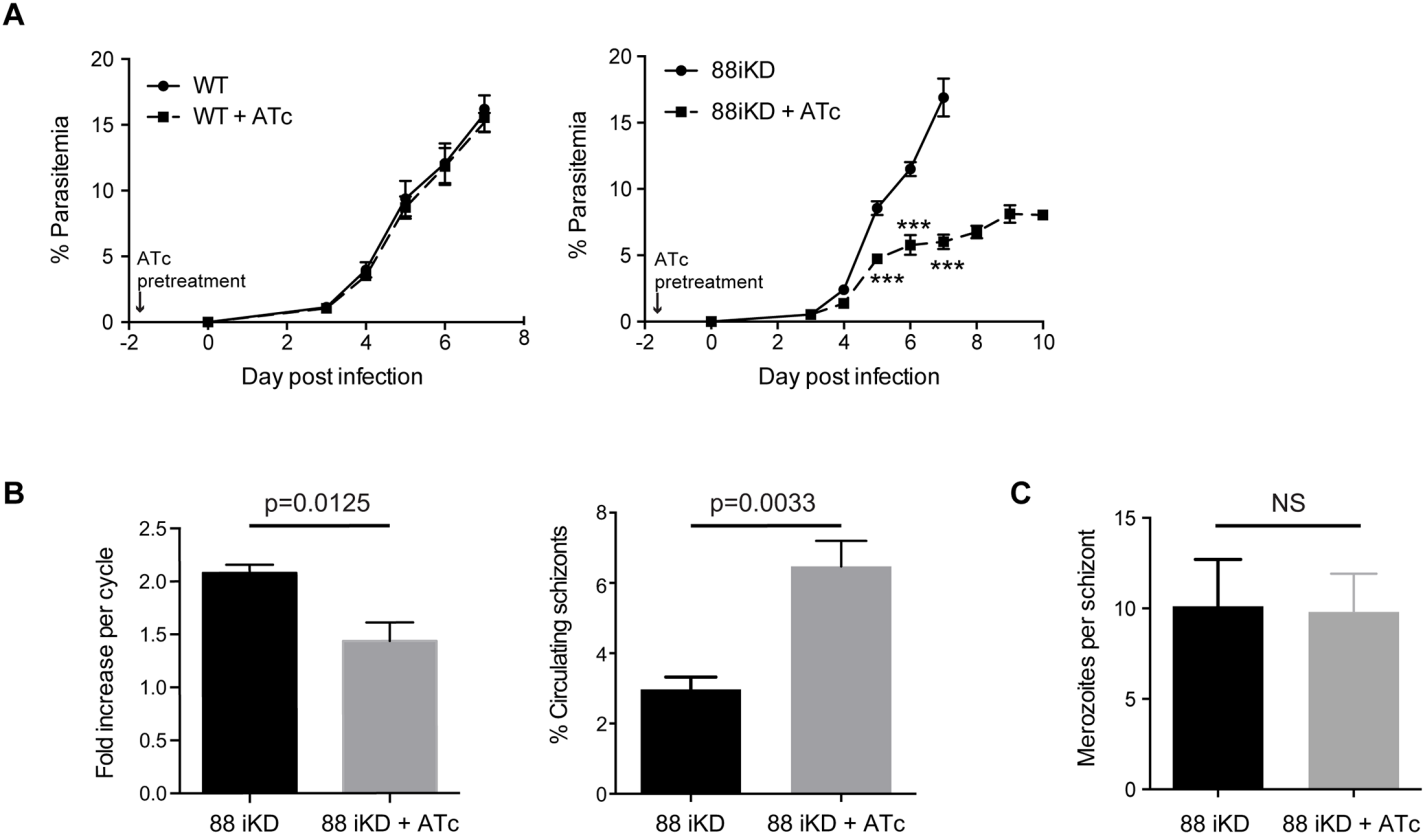
Knockdown of PTEX88 in *P*. *berghei* impacts growth of *P*. *berghei in vivo*. **A**: Parasitemia of Balb/c mice administered either ATc (dashed line) or vehicle control (solid line) after intraperitoneal administration of asynchronous PbANKA wildtype (WT) parasites (left panel) or PbPTEX88 iKD parasites (right panel). Each data point represents the mean ± SEM, n = 6 mice per group. ***P<0.001 as determined by unpaired *t*-test. **B**: Conditional depletion of PTEX88 leads to a lower fold-increase in parasitemia (left panel) and increase in circulating schizonts (right panel) in synchronous infections initiated by intravenous injection of merozoites. **C**: Conditional depletion of PTEX88 does not impact on merozoite formation within schizonts (n = 25).

We next analysed whether the subtle impact on protein export affected the ability of *P*. *berghei* conditionally depleted of PTEX88 to robustly grow in mice and be pathogenic. In the first experiments, Balb/c mice were pre-treated with ATc or vehicle control prior to intraperitoneal infection with asynchronous PbPTEX88 iKD parasites or wildtype PbANKA parasites as a control. Whilst wildtype parasites were unaffected by the presence of ATc, there was a clear and significant reduction in the parasitemias of mice infected with PbPTEX88 iKD and exposed to ATc (Fig 6A). This finding is consistent with the significant delay in growth of *ptex88* null parasites when compared to wild type parasites [34].

It was reasoned that delay of parasite growth could be attributable to one of the following factors, these being fewer merozoites produced per cycle leading to fewer invasion events, an increase in the length of cell cycle resulting in fewer parasite generations over time, or increased parasite clearance. To examine these possibilities, synchronous infections of ATc and vehicle-treated mice were established by intravenous inoculation with PbPTEX88 iKD merozoites and parasites were either followed for two cycles *in vivo* whilst synchronicity was preserved, or harvested at 12 hpi and then cultured *in vitro* in the presence or absence of ATc for the remainder of the cell cycle to enable visualization of older parasites that normally sequester *in vivo*. These experiments revealed that newly invaded ring-stage parasites appeared in the circulation of all mice at the same time for both cycles (S1A Fig), but that parasites with reduced levels of PTEX88 showed a 2.5-fold increase in schizonts in the peripheral blood circulation and a lower fold increase in parasitemia from one cycle to the next (Fig 6B). The *in vitro* experiments not only corroborated that there was no difference between the parasites in the length of their cycle (S1B Fig), it also revealed that the number of merozoites generated per schizont were comparable (Fig 6C) as has been similarly observed with *P*. *falciparum* PTEX88 knockdown. Given that purified merozoites from *P*. *berghei* parasites grown in the presence or absence of ATc demonstrated similar capacity to invade erythrocytes across three independent experiments and that no invasive phenotype was observed when PTEX88 expression was knocked down in *P*. *falciparum*, this points to knockdown of PTEX88 leading to increased parasite clearance from the circulation.

**Fig 7:**
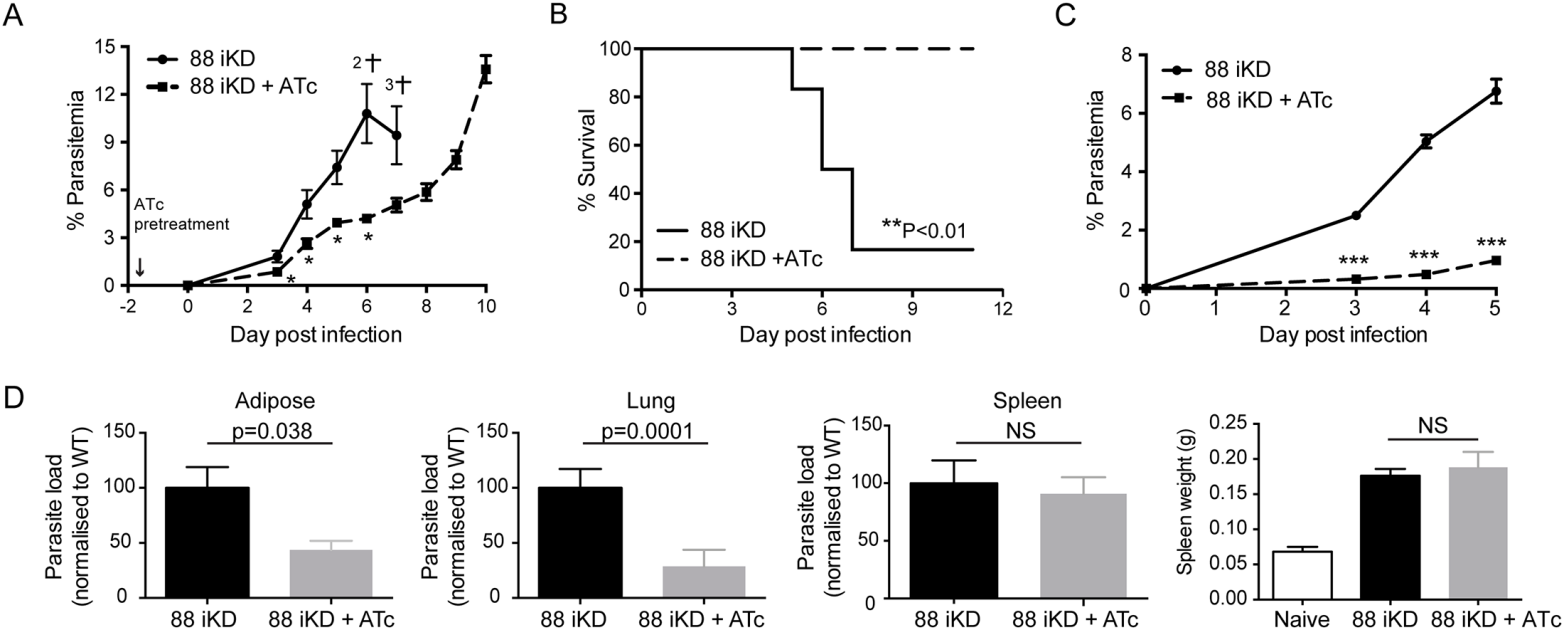
Conditional depletion of PTEX88 affects parasite burden and virulence. **A, C**: Parasitemia and **B**: Survival curves of C57/Bl6 mice administered either ATc (dashed lines) or vehicle control (solid lines) after intraperitoneal administration of 1x10^6^ PbPTEX88 iKD parasites. Crosses represent the number of deaths. *P<0.05, **P<0.01, ***P<0.001 as determined by unpaired *t*-test for parasitemias or by log-rank test for survival curves, which plots the % of mice that have not succumbed to cerebral malaria. **D**: The parasite load in tissues of mice from **C** was determined by normalizing the expression levels of parasite *18S ribosomal RNA* against the mouse *hrpt* house-keeping gene.

Further investigations of the PbPTEX88 iKD parasites were performed using C57/Bl6 mice to determine the consequences of conditional depletion of PTEX88 on parasite burden and virulence. Knockdown of PTEX88 caused a comparable growth delay in C57/Bl6 mice (Fig 7A) as observed in Balb/c mice (Fig 6A) but strikingly, no C57/Bl6 mice treated with ATc succumbed to cerebral malaria (Fig 7B). Instead these mice developed higher parasitemias. These experiments were repeated on a further three occasions but this time mice were humanely culled at day 5 prior to the onset of cerebral malaria so that parasite load could be determined in the adipose tissue, lung and spleen. In the first of these experiments, when knockdown of PTEX88 led to quite a pronounced affect on parasite growth in the circulation (Fig 7C), the parasite load in the adipose tissue and lung was significantly reduced, yet the load in the spleen and the spleen weight was comparable to parasites expressing PTEX88 (Fig 7D). In the second experiment, the inoculum of parasites that had been conditionally depleted of PTEX88 was increased 50-fold (to 5x10^7^ parasites) above that of the vehicle control group so that on the day of harvest the parasitemias were similar (S2A Fig). The loads of parasites with depleted PTEX88 in the adipose tissue and lung were still lower when compared to parasites expressing wildtype levels of PTEX88 although the difference was no longer significant but this time parasite loads in the spleen were significantly higher (S2B Fig). In the third experiment, the knockdown of PTEX88 resulted in similar parasitemias to what had been observed in Fig 7B where mice did not succumb to cerebral malaria (see S3A Fig for comparison between experiments). Again, despite lower parasite numbers in the circulation, parasites grown in the presence of ATc exhibited a similar load in the spleen compared to parasites expressing wildtype levels of PTEX88.

**Fig 8:**
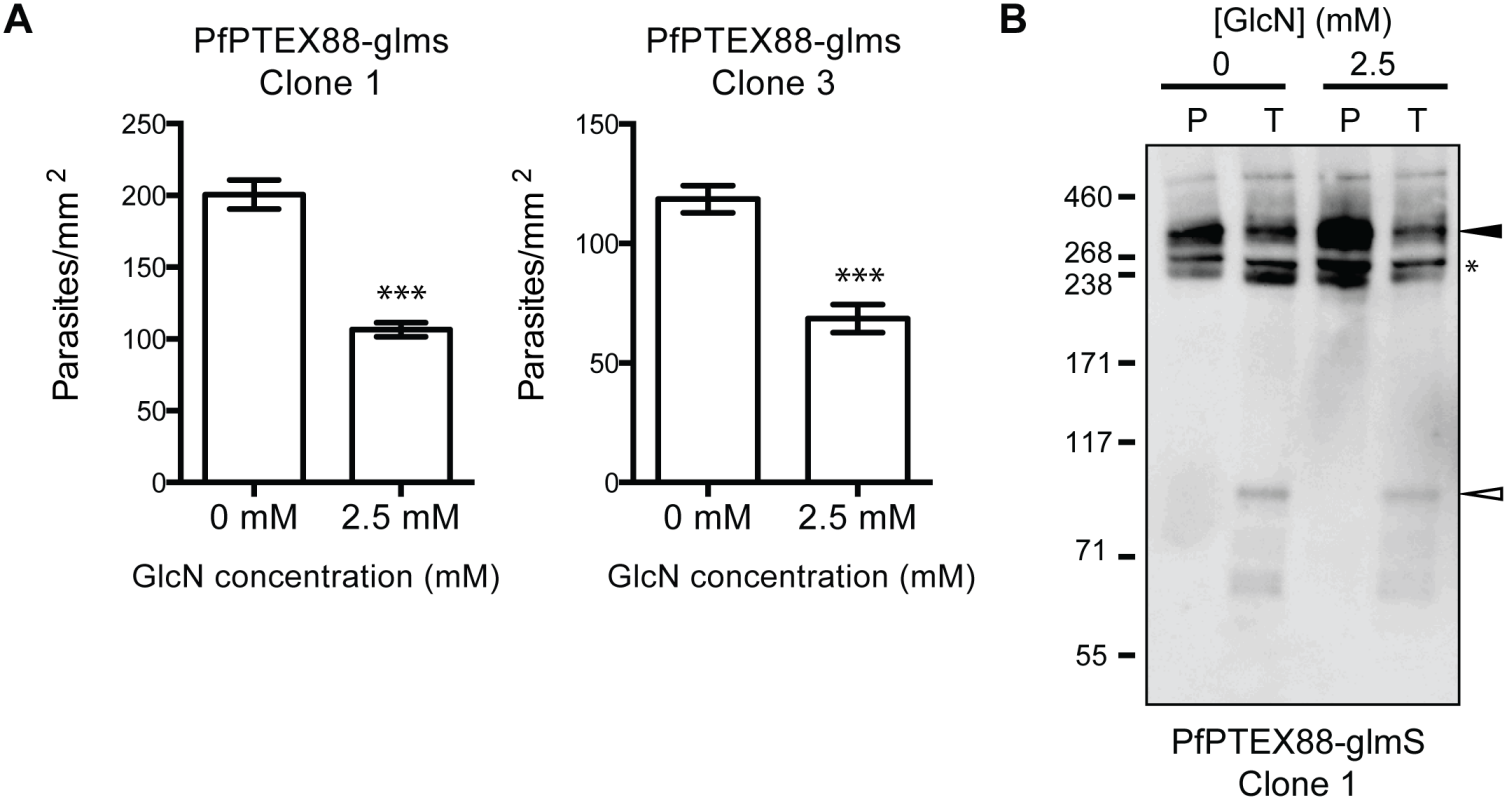
PTEX88-glmS parasites exposed to glucosamine export PfEMP1 but are less cytoadherent to CD36. **A**: Adherence of trophozoite-stage infected erythrocytes to recombinant CD36 under flow conditions (0.1 Pa) ± SEM, n = 30 (***, P<0.001), unpaired t-test. **B**: Trypsin digestion of surface exposed PfEMP1 in erythrocytes infected with PTEX88-glmS parasites grown in the presence and absence of glucosamine (GlcN). P: pre-treated, T: trypsin treated. Full-length PfEMP1 (~270 kDa, black arrowhead) and a cross-reactive spectrin band (~240 kDa, asterisks) are indicated. Trypsin cleavage products (75 kDa) are indicated with an empty arrowhead.

As PbPTEX88 knockdown parasites exhibited an increase in the number of schizonts in the circulation and lower parasite burdens in the peripheral tissues we reasoned that a reduction in cytoadherence of infected erythrocytes to the microvasculature endothelium was the most likely cause of increased parasite clearance. To investigate this further, trophozoite stage parasites were analysed for their ability to cytoadhere to recombinant CD36. These experiments were performed with PTEX88-glmS parasites *in vitro* as both *P*. *falciparum* 3D7 and *P*. *berghei* ANKA cytoadhere to CD36 but *P*. *falciparum* trophozoites are more accessible for study. These experiments revealed that PTEX88-glmS parasites grown in the presence of 2.5 mM GlcN showed a significant reduction in their ability to cytoadhere to CD36 (Fig 8A) with similar results observed for multiple PTEX88-glmS clones. This defect was observed despite the fact that similar levels of PfEMP1 were present in the Maurer's clefts and at the erythrocyte surface, as both IFA and trypsin cleavage of proteins on the surface of infected erythrocytes revealed that PfEMP1 could still be exported (Fig 2F) and presented on the erythrocyte surface (Fig 8B) when PTEX88 was depleted. Notably, *P*. *berghei* also lack PfEMP1 and so it remains to be elucidated what protein(s) mediate parasite cytoadherence and sequestration.

### *P*. *berghei* PTEX88 knockdown parasites still generate a robust pro-inflammatory immune response

In addition to assessing parasite load in the third experiment described above, cytokine and chemokine analysis of the spleen and serum was undertaken and the proportion of splenic cell populations was ascertained to determine whether knocking down PTEX88 influenced the generation of an immune response. These experiments revealed that there was no significant difference in the levels of 26 cytokines or chemokines in the spleens and serum between ATc and vehicle-control treated mice, with the only exception being IL-6, which was significantly increased in the serum of mice infected with parasites conditionally depleted of PTEX88 (S3B Fig and data not shown). With respect to cell proportions in the spleen, there were no significant differences in dendritic cell (DC) subsets (conventional versus plasmacytoid), CD4^+^ or CD8^+^ T cells, B cells, NK cells or neutrophils (data not shown). Moreover, measurement of MHC Class II, CD86 or CD40 expression on CD8^+^ or CD8^-^ conventional DC did not reveal a difference in their activation status (data not shown). There was, however, a significant increase of CD40 expression on plasmacytoid DCs in spleens of mice treated with ATc, a surprising finding given that this subset of DCs is not known to present parasite antigens to T cells during a blood-stage infection. The only other notable difference in the spleen between the two treatment groups was a significantly higher proportion of CD11c^+^MHCII^+^ DCs, CD11c^low^CD11b^high^Ly6C^+^CD64^+^ macrophages, and Ly6C^high^ monocytes (which typically traffic to the sites of infection or inflammation and differentiate into classical macrophages that secrete pro-inflammatory cytokines) and a significantly lower proportion of Ly6C^low^ monocytes (secrete anti-inflammatory cytokines) in mice infected with parasites conditionally depleted of PTEX88 (S3C Fig).

## Discussion

In this study we were able to compare and contrast the effects of conditionally regulating PTEX88 under *in vitro* and *in vivo* conditions to gain insight into PTEX88 function. Knockdown of PTEX88 in both *P*. *falciparum* and *P*. *berghei* did not influence parasite maturation in erythrocytes, nor the length of the cell cycle. However, the ability to analyse *P*. *berghei* in an *in vivo* setting revealed that overall growth of parasites was impacted, stemming from a lower-fold increase in parasitemia from one cycle to the next. The impact became more pronounced after the first few days of infection *in vivo*. We could rule out that knocking down PTEX88 has an effect on invasion as previously put forward by Matz et al [34] in their *P*. *berghei* PTEX88 knockout studies, as the schizont to ring transition in *P*. *falciparum* was unaffected. Instead, the increase in schizonts in the circulation of *P*. *berghei*-infected mice led us to speculate that the observed reduction in growth caused by PTEX88 depletion was due an increased clearance of these parasites by the spleen and hence a lower number of schizonts rupturing in the circulation and giving rise to a new cycle of infection. In keeping with this, *P*. *falciparum* parasites depleted of PTEX88 exhibited a reduced ability to adhere to CD36 and the load of *P*. *berghei* PTEX88 knockdown parasites in the spleen was similar or higher than parasites expressing wildtype levels of PTEX88, despite the fact that the load in the adipose tissue and lung were reduced. Of all the splenic cell populations that increased, it was the CD11c^+^MHCII^+^ DCs, CD11c^low^CD11b^high^Ly6C^+^CD64^+^ macrophages, and Ly6C^high^ monocytes, consistent with the non-sequestering schizonts in the spleen being cleared through phagocytosis. We did not observe the splenomegaly reported with null *ptex88*^-^ parasites [34], however, it should be noted that as it is not possible to fully eliminate PTEX88 expression with the transcriptional ATc-regulation system, the effect on sequestration and hence clearance through the spleen would be less pronounced.

Surprisingly, however, the level of PTEX88 knockdown was still sufficient to lead to a profound defect in the ability of these parasites to cause cerebral malaria. This was unexpected because the PTEX88 depleted parasites were still able to induce a robust pro-inflammatory cytokine and chemokine response. In the study by Matz et al [34], the ability of the null *ptex88*^-^ parasites to elicit a pro-inflammatory response was not investigated. Intriguingly, however, whilst mice infected with null *ptex88*^-^ parasites exhibited reduced sequestration in the brain and did not succumb to cerebral malaria, their brains were histologically indistinguishable from those taken from mice infected with wildtype parasites with respect to cerebral bleeding and activation of microglia and astrocytes, indicative of an inflammatory response [34]. Although we did not observe a difference in the proportion of immune cells in the spleen in mice infected with wildtype or depleted levels of PTEX88, it would be worthwhile undertaking further studies to examine whether the differences in the pathology of the two groups of mice is the result of reduced infiltration of CD8^+^ T cell numbers in the brain, particularly as these cells are known to contribute to the induction of cerebral malaria in experimental murine models [46].

As PTEX88 has been shown using various immunoprecipitation approaches to be a component of the protein export machinery [24,27], the effect of knocking down PTEX88 on protein export was additionally examined in this study. In stark contrast to PTEX150 and HSP101, where a striking defect in protein export was observed when these components were conditionally depleted [25,26], export of a variety of PEXEL and PNEP proteins was not affected after conditional depletion of PTEX88 in either *P*. *falciparum* or *P*. *berghei*. It is important to note that other translocation systems in nature comprise auxiliary components that are not essential for growth or protein translocation under particular conditions. For instance, the Sec translocon in prokaryotes and eukaryotes is modular and engages additional subunits and partner proteins depending on the nature of the substrate to be translocated [47]. The peroxisomal translocon also harbours two distinct receptors, Pex5 and Pex7 that act as receptors for specific subsets of cargo [48]. There are several possible ways to interpret how deletion of the various PTEX components can lead to different export phenotypes. PTEX may recruit PTEX88 to facilitate the export of proteins (for example by contributing to protein unfolding or quality control) or a subset of cargo that either directly or indirectly influences sequestration. However, the ligands that mediate sequestration in *P*. *berghei* are currently not known and the caveat with revealing their identity with the strategies used herein relies on having specific reagents to these proteins to study them. At this stage we can rule out that PTEX88 is serving as a PTEX receptor/adaptor for PfEMP1 as PTEX88-glmS parasites treated with glucosamine still express PfEMP1 on the infected erythrocyte surface and *P*. *berghei* lack PfEMP1. A comparison of proteins that traffic to the erythrocyte surface when PTEX88 is depleted from *P*. *falciparum* and *P*. *berghei* would be the most direct way to determine whether the same protein(s) is responsible for the loss of sequestration of *P*. *berghei in vivo* and cytoadhesion to CD36 *in vitro* or whether the cytoadhesive effect is an indirect one. It is also important to bear in mind that the role of PTEX88 may only become prominent *in vivo* under particular conditions faced by the parasite and hence examination of protein export in *P*. *falciparum* cultured *in vitro* will fail to reveal this role. In mitochondria, for example, the Tim8p-Tim13p complex is recruited to the mitochondrial inner membrane to facilitate protein translocation specifically during periods of low membrane potential [49]. It may also be the case that the acute differences in protein export upon depletion of PTEX88 are not detectable with the available tools for measuring protein export yet the cumulative effect is sufficient to give rise to defects in cytoadherence and virulence. For example, many exported proteins contribute to skeletal remodeling of *P*. *falciparum*-infected erythrocytes and the proper trafficking, folding and presentation of PfEMP1 on the erythrocyte surface. As a combination of skeletal remodeling and deposition of knobs accounts for dramatic increases in rigidity and facilitates adhesion to endothelial cell ligands by distributing the tensional forces imposed on individual PfEMP1 molecules across the entire knob region and through to the cytoskeleton [50], subtle changes to the quantity, timing or folding of exported proteins could alter the overall impact on the cytoadhesive force. The development of more complex screening methods to look at the whole exportome may, therefore, reveal more about a role for PTEX88 in protein export.

In contrast to the PTEX88 knockdown parasites, those parasites in which *trx2* has been genetically deleted exhibited a growth delay across the asexual blood stage life cycle, already apparent during the ring stages when protein export normally commences. Whilst the *trx2* knockout parasites displayed fewer parasite proteins on the erythrocyte surface compared to erythrocytes infected with wildtype parasites, the impact on parasite virulence was much less profound than with the PTEX88 knockdown parasites [27]. This suggests that TRX2 plays a role in regulating export of a wider array of proteins compared to PTEX88. Thus while PTEX88 and TRX2 are both auxiliary components of PTEX, it is likely they serve different roles in protein translocation across the PVM.

## Supporting Information

S1 Fig Depletion of PTEX88 in *P*. *berghei* reduces parasite amplification *in vivo* but not via extension of the cell cycle. **A**. Representative growth curves of PbPTEX88 iKD parasites grown in the presence or absence of ATc from two independent experiments initiated by intravenous injection of purified merozoites. The appearance of new ring stages in Giemsa smears indicates that invasion had occurred and hence the length of previous cycle. **B**: *In vitro* growth analysis of PbPTEX88 iKD parasites. Infection was initiated by intravenous injection of purified merozoites into mice and at 9 hpi, parasites were harvested and cultured *in vitro* for the remainder of the cell cycle. ER, early ring; LR, late ring; ET, early trophozoite; LT, late trophozoite; ES, early schizont; LS, late schizont. A minimum of 100 parasitised cells was counted for each timepoint.
(TIF)

S2 Fig Depletion of PTEX88 leads to lower parasite burden in adipose tissue and lung but an increase in parasite load in the spleen. **A**: Parasitemia of C57/Bl6 mice administered either ATc (dashed lines) or vehicle control (solid lines) after intraperitoneal administration of 5x10^7^ or 1x10^6^ PbPTEX88 iKD parasites, respectively. By day 5 post-infection, when tissues were harvested to assess parasite load, the blood parasitemia between the two lines was equivalent. **B**: The parasite load in tissues was determined by normalizing the expression levels of *parasite 18S ribosomal RNA* against the mouse *hrpt* house keeping gene.
(TIF)

S3 Fig Knockdown of PTEX88 does not impact on the ability of the parasite to generate a robust pro-inflammatory response. **A**: Parasitemia of C57/Bl6 mice (n = 5) administered either ATc (dashed lines) or vehicle control (solid lines) after intraperitoneal administration of 1x10^6^ PbPTEX88 iKD parasites. The lower panel shows the parasitemia curves on mice from which the cerebral malaria studies were performed as a comparison. **B**: Parasite load in tissues harvested at day 5 post-infection from mice in A, upper panel. **C**: Graphs showing the spleen cyotokine and chemokine levels of mice infected with parasites with wildtype (-ATc) or depleted PTEX88 (+ATc) expression. Shown are the plots for IL-6, IL-10, IL-18, TNF-**α** and IFN-.**γ**Additional cytokines and chemokines for which plots are not shown include GM-CSF, IL-1b, IL12p70, IL-2, IL-4, IL-5, IL-9, IL13, IL17, IL-22, Eotaxin, CXCL1, IP10, MCP-1, MCP-3, MIP1a, MIP1b, MIP2 and Rantes. **D**: Graphs showing cell populations in the spleen at day 5 post-infection (means ± SD).
(TIF)

S1 Table Oligonucleotides used in this study. (DOCX)
