# Supplementary figures and images for: Contrasting Inducible Knockdown of the Auxiliary PTEX Component PTEX88 in P. falciparum and P. berghei Unmasks a Role in Parasite Virulence
Source: PLoS One. 2016 Feb 17;11(2):e0149296. doi: 10.1371/journal.pone.0149296 (PMC4757573; doi:10.1371/journal.pone.0149296)

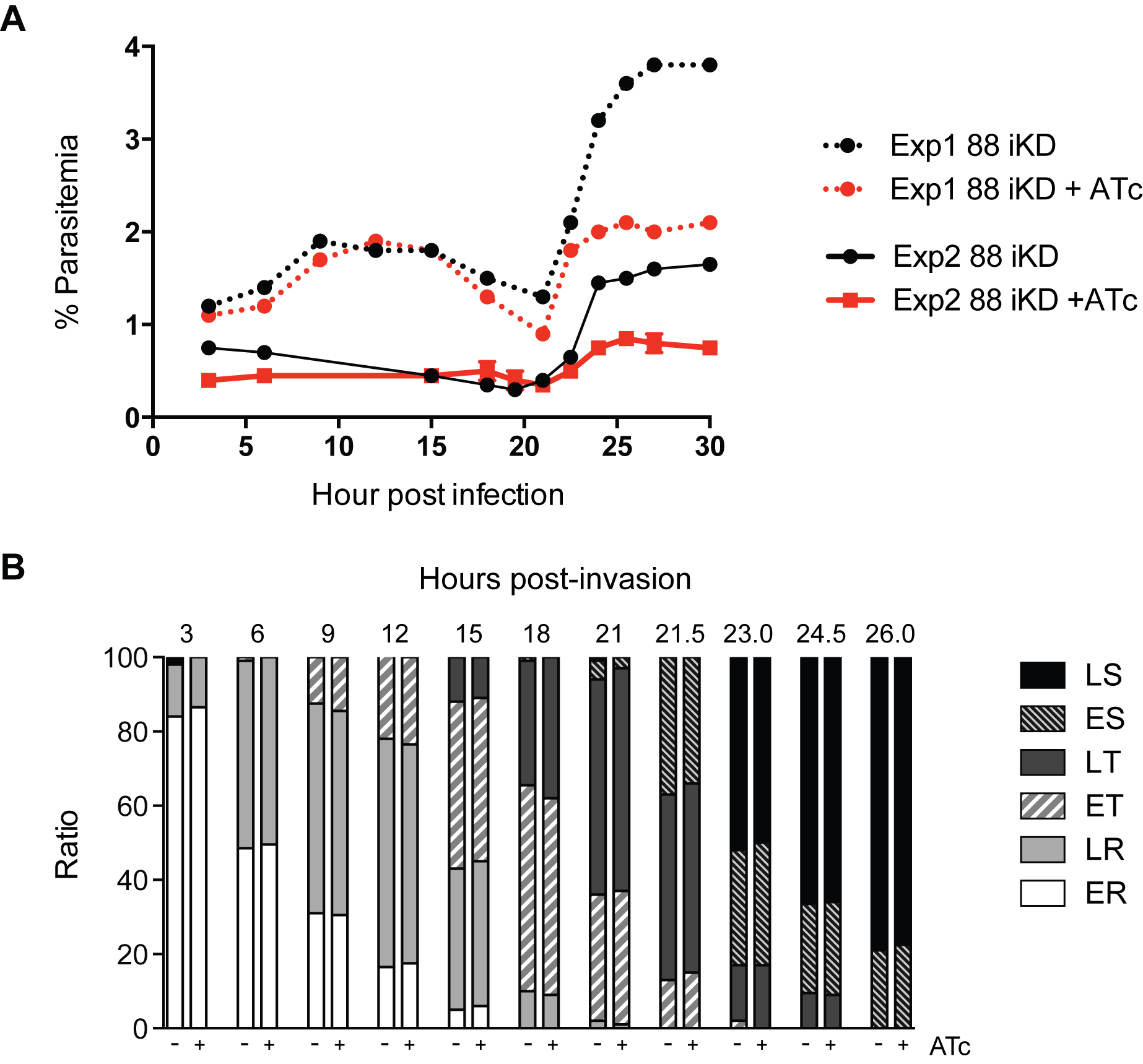

Supplement: S1 Fig — A. Representative growth curves of PbPTEX88 iKD parasites grown in the presence or absence of ATc from two independent experiments initiated by intravenous injection of purified merozoites. The appearance of new ring stages in Giemsa smears indicates that invasion had occurred and hence the length of previous cycle. B: In vitro growth analysis of PbPTEX88 iKD parasites. Infection was initiated by intravenous injection of purified merozoites into mice and at 9 hpi, parasites were harvested and cultured in vitro for the remainder of the cell cycle. ER, early ring; LR, late ring; ET, early trophozoite; LT, late trophozoite; ES, early schizont; LS, late schizont. A minimum of 100 parasitised cells was counted for each timepoint. (TIF) [file pone.0149296.s001.tif]

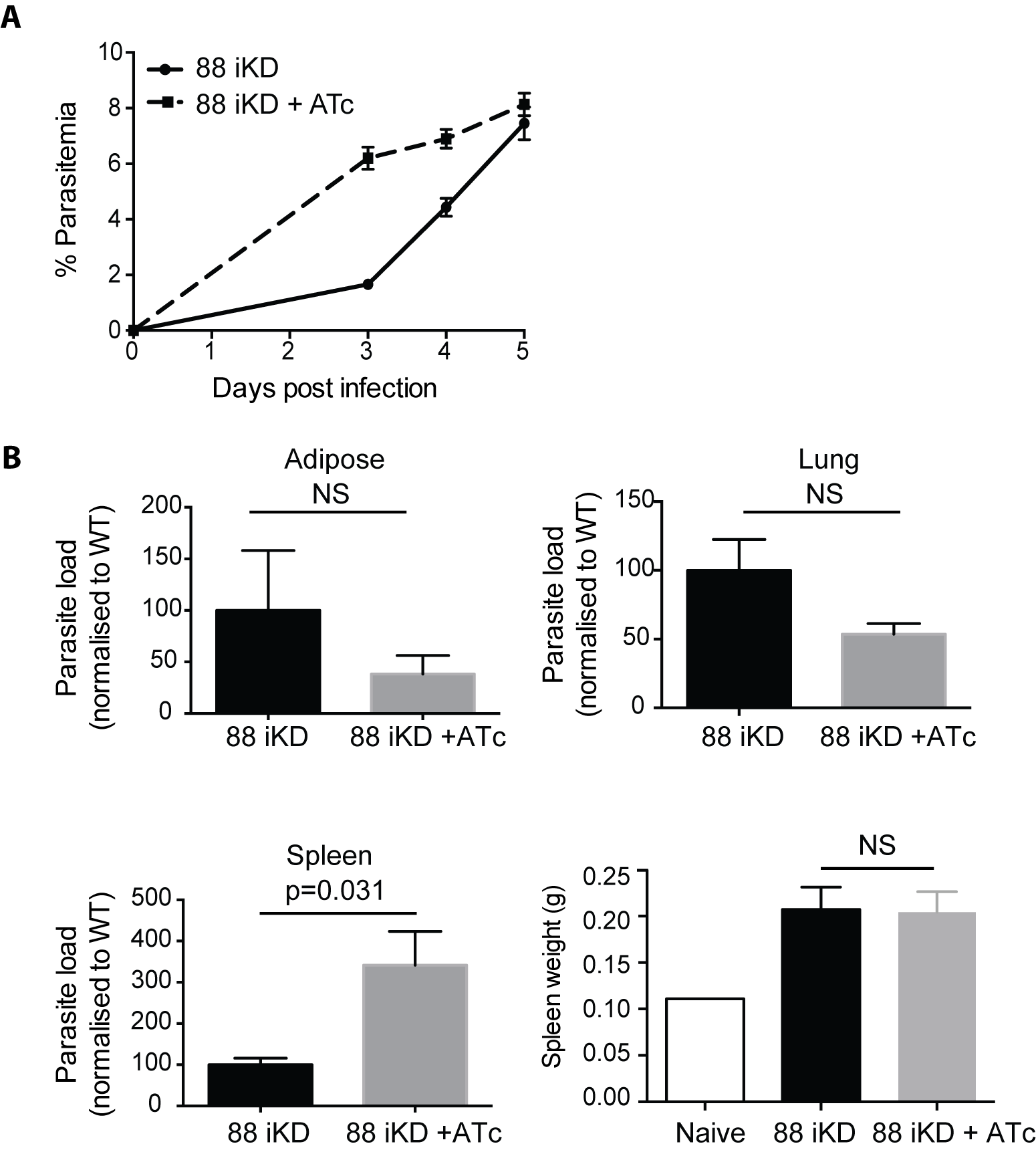

Supplement: S2 Fig — A: Parasitemia of C57/Bl6 mice administered either ATc (dashed lines) or vehicle control (solid lines) after intraperitoneal administration of 5x10^7^ or 1x10^6^ PbPTEX88 iKD parasites, respectively. By day 5 post-infection, when tissues were harvested to assess parasite load, the blood parasitemia between the two lines was equivalent. B: The parasite load in tissues was determined by normalizing the expression levels of parasite 18S ribosomal RNA against the mouse hrpt house keeping gene. (TIF) [file pone.0149296.s002.tif]

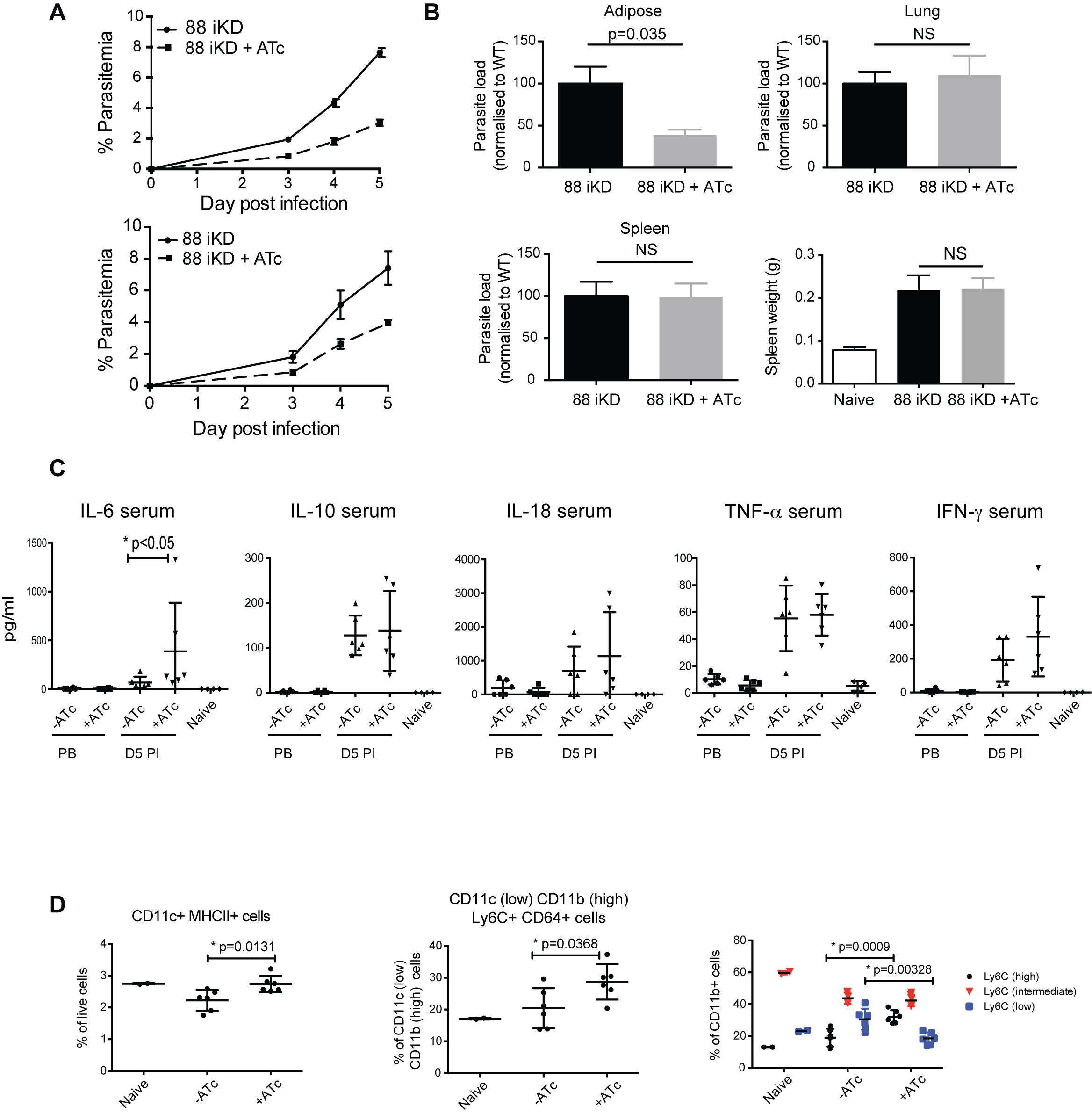

Supplement: S3 Fig — A: Parasitemia of C57/Bl6 mice (n = 5) administered either ATc (dashed lines) or vehicle control (solid lines) after intraperitoneal administration of 1x10^6^ PbPTEX88 iKD parasites. The lower panel shows the parasitemia curves on mice from which the cerebral malaria studies were performed as a comparison. B: Parasite load in tissues harvested at day 5 post-infection from mice in A, upper panel. C: Graphs showing the spleen cyotokine and chemokine levels of mice infected with parasites with wildtype (-ATc) or depleted PTEX88 (+ATc) expression. Shown are the plots for IL-6, IL-10, IL-18, TNF-α and IFN-.γAdditional cytokines and chemokines for which plots are not shown include GM-CSF, IL-1b, IL12p70, IL-2, IL-4, IL-5, IL-9, IL13, IL17, IL-22, Eotaxin, CXCL1, IP10, MCP-1, MCP-3, MIP1a, MIP1b, MIP2 and Rantes. D: Graphs showing cell populations in the spleen at day 5 post-infection (means ± SD). (TIF) [file pone.0149296.s003.tif]
