# Supplementary material for: Contrasting Inducible Knockdown of the Auxiliary PTEX Component PTEX88 in P. falciparum and P. berghei Unmasks a Role in Parasite Virulence
Source: PLoS One. 2016 Feb 17;11(2):e0149296. doi: 10.1371/journal.pone.0149296 (PMC4757573; doi:10.1371/journal.pone.0149296)
Supplement: S1 Table — (DOCX) [file pone.0149296.s004.docx]

**S1Table. Oligonucleotides used in this study**

| **Oligo Name** | **Sequence (5' – 3')** | **Gene** |
| --- | --- | --- |
| SH4R | gccgaattcCCCATCTTTTTTTATTTTCTACCC | *Pb ptex88* 3’UTR |
| O145R | ctcgagAGCGGCATAATCTGGAACATC | Haemagluttinin tag |
| O162R | ccaccggtactagtAAATTGAAGGAAAAACCATCATTTGTG | *trad* |
| O213F | AGTCGAGCTCGGTACCCAATTCGCCC | Inducible promoter |
| O276R | GTGATTTCTCTTTGTTCAAGGA | *glmS* |
| O295F | gccggatccGTGCAGATCATTATTAACAGA | *Pb ptex88* CDS |
| O451F | gtgctgcagATGATGCTTTATTTTATTGTGT | *Pb ptex88* CDS |
| O452R | gacgctagcATTTATCTATCGTTAATCCAGTG) | *Pb ptex88* CDS |
| O453F | gacgctagcTTGTAATATTCGGGCTTTAAGTAA | *Pb ptex88* 5’ UTR |
| O454R | tgagcgcgcAATCGAATTTTGGGGATTTCAA | *Pb ptex88* 5’ UTR |
| O587F | TTTTATTGTGTTGGCATTGG | *Pb ptex88* |
| O588R | CGGGAAGTTCATCAAGTATT | *Pb ptex88* |
| O567F | AATTAAAGAAGCATCTGAGGGTCCAC | *Pb gapdh* |
| O568R | TTGAATATCCCCATTCATTGTCATACC | *Pb gapdh* |
| O629F | AAGCATTAAATAAAGCGAATACATCCTTAC | *Pb 18S rRNA* |
| O630R | GGAGATTGGTTTTGACGTTTATGTG | *Pb 18S rRNA* |
| O631F | CATTATGCCGAGGATTTGGA | *Mouse hprt* |
| O632R | AATCCAGCAGGTCAGCAAAG | *Mouse hprt* |
